# *N*-(4-Chloro-1,3-benzothia­zol-2-yl)-2-(3-methyl­phen­yl)acetamide monohydrate

**DOI:** 10.1107/S1600536811035872

**Published:** 2011-09-14

**Authors:** A. S. Praveen, Jerry P. Jasinski, James A. Golen, H. S. Yathirajan, B. Narayana

**Affiliations:** a Department of Studies in Chemistry, University of Mysore, Manasagangotri, Mysore 570 006, India; b Department of Chemistry, Keene State College, 229 Main Street, Keene, NH 03435-2001, USA; c Department of Studies in Chemistry, Mangalore University, Mangalagangotri, 574 199, India

## Abstract

In the title compound, C_16_H_13_ClN_2_OS·H_2_O, the dihedral angle between the mean planes of the benzothia­zole ring system and the methylphenyl ring is 79.3 (6)°. The crystal packing features inter­molecular O—H⋯N, O—H⋯O and N—H⋯O hydrogen bonds involving the water mol­ecule and weak C—H⋯O, C—H⋯*Cg* and π–π stacking inter­actions [centroid–centroid distances = 3.8743 (7), 3.7229 (7) and 3.7076 (8) Å].

## Related literature

For the biological activity of compounds with benzothia­zole skeletons, see: Aiello *et al.* (2008 ▶); Cho *et al.* (2008 ▶). For their structural similarity to the lateral chain of natural benzyl­penicillin, see: Mijin & Marinkovic (2006 ▶); Mijin *et al.* (2006 ▶, 2008 ▶) and for their coordination abilities, see: Wu *et al.* (2008 ▶, 2010 ▶). For related structures, see: Davis & Healy (2010 ▶); John *et al.* (2010 ▶); Nogueira *et al.* (2010 ▶); Praveen *et al.* (2011 ▶); Selig *et al.* (2010 ▶); Wen *et al.* (2010 ▶); Xiao *et al.* (2010 ▶). For standard bond lengths, see Allen *et al.* (1987 ▶).
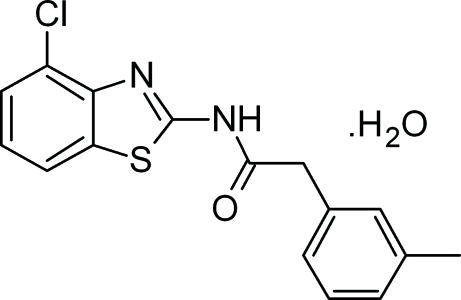


## Experimental

### 

#### Crystal data

C_16_H_13_ClN_2_OS·H_2_O
*M* *_r_* = 334.81
Triclinic, 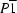

*a* = 7.2771 (3) Å
*b* = 9.2568 (5) Å
*c* = 12.0851 (5) Å
α = 83.948 (4)°
β = 84.306 (3)°
γ = 72.133 (4)°
*V* = 768.58 (6) Å^3^
*Z* = 2
Mo *K*α radiation
μ = 0.39 mm^−1^
*T* = 173 K
0.25 × 0.21 × 0.20 mm

#### Data collection

Oxford Diffraction Xcalibur Eos Gemini diffractometer
Absorption correction: multi-scan (*CrysAlis RED*; Oxford Diffraction, 2010 ▶) *T*_min_ = 0.908, *T*_max_ = 0.926
10307 measured reflections
4303 independent reflections
3834 reflections with *I* > 2σ(*I*)
*R*_int_ = 0.013

#### Refinement

*R*[*F* ^2^ > 2σ(*F* ^2^)] = 0.031
*wR*(*F* ^2^) = 0.089
*S* = 1.01
4303 reflections
209 parameters
4 restraints
H atoms treated by a mixture of independent and constrained refinement
Δρ_max_ = 0.40 e Å^−3^
Δρ_min_ = −0.28 e Å^−3^

### 

Data collection: *CrysAlis PRO* (Oxford Diffraction, 2010 ▶); cell refinement: *CrysAlis PRO*; data reduction: *CrysAlis RED* (Oxford Diffraction, 2010 ▶); program(s) used to solve structure: *SHELXS97* (Sheldrick, 2008 ▶); program(s) used to refine structure: *SHELXL97* (Sheldrick, 2008 ▶); molecular graphics: *SHELXTL* (Sheldrick, 2008 ▶); software used to prepare material for publication: *SHELXTL*.

## Supplementary Material

Crystal structure: contains datablock(s) global, I. DOI: 10.1107/S1600536811035872/im2316sup1.cif

Structure factors: contains datablock(s) I. DOI: 10.1107/S1600536811035872/im2316Isup2.hkl

Supplementary material file. DOI: 10.1107/S1600536811035872/im2316Isup3.cml

Additional supplementary materials:  crystallographic information; 3D view; checkCIF report

## Figures and Tables

**Table 1:** Hydrogen-bond geometry (Å, °)

*D*—H⋯*A*	*D*—H	H⋯*A*	*D*⋯*A*	*D*—H⋯*A*
O2—H2*OB*⋯N1^i^	0.88 (1)	2.10 (2)	2.924 (1)	158 (2)
O2—H2*OA*⋯O1^ii^	0.88 (1)	2.05 (1)	2.904 (1)	164 (2)
N2—H2*N*⋯O2	0.87 (1)	1.92 (1)	2.785 (1)	177 (2)
C5—H5*A*⋯O1^iii^	0.95	2.56	3.351 (2)	141
C3—H3*A*⋯*Cg*3^iv^	0.95	2.66	3.502 (1)	148

Symmetry codes: (i) 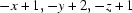; (ii) 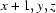; (iii) 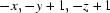; (iv) 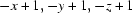.

**Fig. 1.**
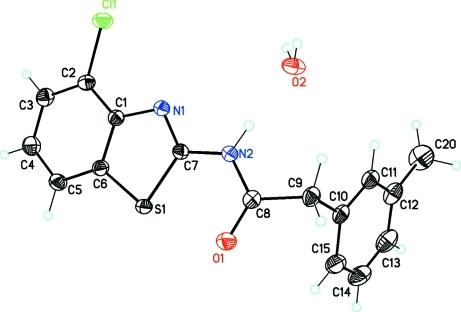
Molecular structure of the title compound showing the atom labeling scheme and 30% probability displacement ellipsoids.

**Fig. 2.**
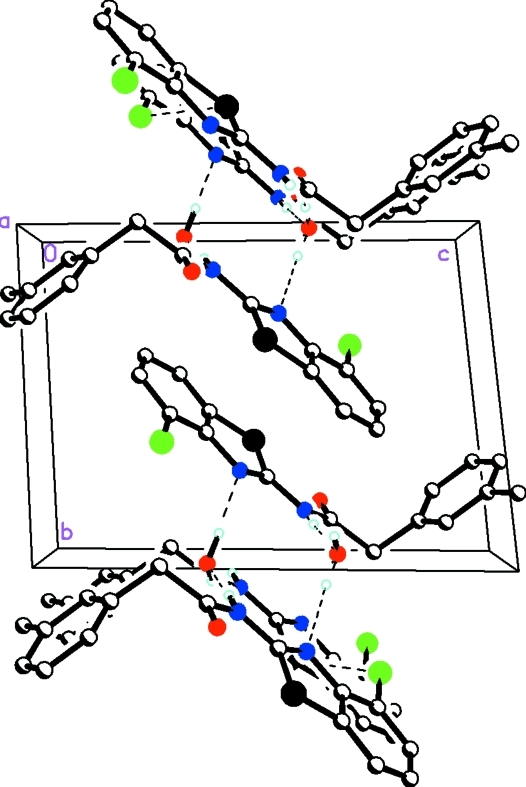
Packing diagram of the title compound viewed down the a axis. Dashed lines indicate O—H···N hydrogen bonds.

## supplementary crystallographic information

### Comment

The biological activity of compounds with benzothiazole skeletons includes anticancer, antibacterial, antifungal and anthelmintic properties (Aiello *et al.*, 2008; Cho *et al.*, 2008) N-Substituted 2-arylacetamides are very interesting compounds because of their structural similarity to the lateral chain of natural benzylpenicillin (Mijin *et al.*, 2008; Mijin *et al.*, 2006). Amides are also used as ligands due to their excellent coordination abilities (Wu *et al.*, 2008; 2010). Crystal structures of some acetamidederivatives, viz., 2-(4-bromophenyl)-N-(2-methoxyphenyl)acetamide (Xiao *et al.*, 2010), N-benzyl-2-(3-chloro-4-hydroxyphenyl)acetamide (Davis *et al.*, 2010), 2-[(5,7-dibromoquinolin-8-yl)oxy]-N-(2-methoxyphenyl)acetamide (Wen *et al.*, 2010), N-(4-bromophenyl)-2-(2-thienyl)acetamide (Nogueira *et al.*, 2010), N-[4-(benzylsulfamoyl)phenyl]acetamide (John *et al.*, 2010), 2-(4-fluorophenyl)-N-{4-[6-(4-fluorophenyl)-2,3-dihydroimidazo[2,1-b][1,3] thiazol-5-yl]pyridin-2-yl}acetamide (Selig *et al.*, 2010) and N-(3-chloro-4-fluorophenyl)-2-(naphthalen-1-yl)acetamide (Praveen *et al.*, 2011) have been reported. As part of our ongoing studies of amides, the title compound is synthesized and its crystal structure is reported.

In the title hydrated compound, C_16_H_13_ClN_2_OS × H_2_O, the dihedral angle between the mean planes of the benzothiazole and benzenes is 79.3 (6)° (Fig. 1). Crystal packing is realized by O–H···N, O—H···O and N—H···O hydrogen bonds involving the water molecule and weak O—H···O, C—H···O, C—H···Cg (Table 1) and π–π stacking (Table 2) intermolecular interactions (Fig. 2).

### Experimental

To a stirred solution of (3-methylphenyl)acetic acid (1 g, 6.65 mmol), triethylamine (1.34 g, 13.31 mmol) and 4-chloro-1,3-benzothiazol-2-amine (1.27 g, 6.65 mmol) in dichloromethane (10 ml), 1-(3-dimethylaminopropyl)-3-ethylcarbodiimide HCl (1.52 g, 7.93 mmol) was added at 273 K. The reaction mixture was stirred at room temperature for 3 h. After the completion of the reaction, the reaction mixture was poured into ice cold water and the layers were separated. The organic layer was washed with 10% aq. NaHCO_3_ solution (10 ml), brine (10 ml), dried over anhydrous Na_2_SO_4_, filtered and concentrated under vacuum to obtain the crude product which was triturated with ethanol and filtered to afford 1.92 g of the title compound (I) as a white solid in 91 % yield. Single crystals were grown from ethanol by slow evaporation method (m.p.: 397-398 K).

### Refinement

H20A, H20B and H2N were located by a Fourier map and refined isotropically. All other H atoms were placed in their calculated positions and then refined using the riding model with atom—H lengths of 0.95Å (CH), 0.99Å (CH_2_) or 0.98Å (CH_3_). Isotropic displacement parameters for these atoms were set to 1.18-1.21 (CH) 1.20 CH_2_) or 1.51 (CH_3_) times *U*_eq_ of the parent atom.

### Crystal data

**Table:**

C_16_H_13_ClN_2_OS·H_2_O	*Z* = 2
*M**_r_* = 334.81	*F*(000) = 348
Triclinic, *P*1	*D*_x_ = 1.447 Mg m^−^^3^
Hall symbol: -P 1	Mo *K*α radiation, λ = 0.71073 Å
*a* = 7.2771 (3) Å	Cell parameters from 5935 reflections
*b* = 9.2568 (5) Å	θ = 3.2–32.2°
*c* = 12.0851 (5) Å	µ = 0.39 mm^−^^1^
α = 83.948 (4)°	*T* = 173 K
β = 84.306 (3)°	Block, colorless
γ = 72.133 (4)°	0.25 × 0.21 × 0.20 mm
*V* = 768.58 (6) Å^3^	

### Data collection

**Table:**

Oxford Diffraction Xcalibur Eos Gemini diffractometer	4303 independent reflections
Radiation source: Enhance (Mo) X-ray Source	3834 reflections with *I* > 2σ(*I*)
graphite	*R*_int_ = 0.013
Detector resolution: 16.1500 pixels mm^-1^	θ_max_ = 29.6°, θ_min_ = 3.2°
ω scans	*h* = −6→10
Absorption correction: multi-scan (*CrysAlis RED*; Oxford Diffraction, 2010)	*k* = −12→12
*T*_min_ = 0.908, *T*_max_ = 0.926	*l* = −16→16
10307 measured reflections	

### Refinement

**Table:**

Refinement on *F*^2^	Primary atom site location: structure-invariant direct methods
Least-squares matrix: full	Secondary atom site location: difference Fourier map
*R*[*F*^2^ > 2σ(*F*^2^)] = 0.031	Hydrogen site location: inferred from neighbouring sites
*wR*(*F*^2^) = 0.089	H atoms treated by a mixture of independent and constrained refinement
*S* = 1.01	*w* = 1/[σ^2^(*F*_o_^2^) + (0.0502*P*)^2^ + 0.2662*P*] where *P* = (*F*_o_^2^ + 2*F*_c_^2^)/3
4303 reflections	(Δ/σ)_max_ = 0.007
209 parameters	Δρ_max_ = 0.40 e Å^−^^3^
4 restraints	Δρ_min_ = −0.28 e Å^−^^3^

### Special details

**Table:**

Geometry. All esds (except the esd in the dihedral angle between two l.s. planes) are estimated using the full covariance matrix. The cell esds are taken into account individually in the estimation of esds in distances, angles and torsion angles; correlations between esds in cell parameters are only used when they are defined by crystal symmetry. An approximate (isotropic) treatment of cell esds is used for estimating esds involving l.s. planes.
Refinement. Refinement of *F*^2^ against ALL reflections. The weighted *R*-factor *wR* and goodness of fit *S* are based on *F*^2^, conventional *R*-factors *R* are based on *F*, with *F* set to zero for negative *F*^2^. The threshold expression of *F*^2^ > σ(*F*^2^) is used only for calculating *R*-factors(gt) *etc*. and is not relevant to the choice of reflections for refinement. *R*-factors based on *F*^2^ are statistically about twice as large as those based on *F*, and *R*- factors based on ALL data will be even larger.

### Fractional atomic coordinates and isotropic or equivalent isotropic displacement parameters (Å^2^)

**Table:**

	*x*	*y*	*z*	*U*_iso_*/*U*_eq_	
S1	0.10411 (4)	0.65402 (3)	0.47845 (2)	0.02017 (8)	
Cl1	0.78434 (4)	0.64763 (4)	0.28146 (3)	0.02807 (9)	
O1	−0.10370 (13)	0.84965 (11)	0.62242 (8)	0.02685 (19)	
O2	0.47923 (14)	0.98584 (11)	0.64369 (9)	0.0289 (2)	
H2OB	0.477 (2)	1.0762 (16)	0.6127 (14)	0.035*	
H2OA	0.600 (2)	0.9272 (17)	0.6380 (15)	0.035*	
N1	0.41640 (14)	0.74054 (11)	0.44287 (8)	0.01894 (19)	
N2	0.19872 (14)	0.86818 (11)	0.58166 (8)	0.01959 (19)	
H2N	0.285 (2)	0.9074 (18)	0.5985 (13)	0.024*	
C1	0.43805 (16)	0.62472 (13)	0.37390 (9)	0.0181 (2)	
C2	0.59653 (16)	0.56742 (13)	0.29843 (10)	0.0202 (2)	
C3	0.60131 (18)	0.44952 (14)	0.23612 (11)	0.0242 (2)	
H3A	0.7097	0.4102	0.1856	0.029*	
C4	0.44702 (19)	0.38796 (14)	0.24728 (11)	0.0263 (3)	
H4A	0.4525	0.3063	0.2043	0.032*	
C5	0.28611 (18)	0.44326 (14)	0.31952 (11)	0.0237 (2)	
H5A	0.1808	0.4018	0.3261	0.028*	
C6	0.28419 (17)	0.56185 (13)	0.38211 (9)	0.0195 (2)	
C7	0.25131 (16)	0.76327 (13)	0.50223 (9)	0.0179 (2)	
C8	0.02371 (17)	0.90296 (13)	0.64119 (10)	0.0200 (2)	
C9	0.00054 (18)	1.00506 (14)	0.73487 (10)	0.0224 (2)	
H9A	−0.1334	1.0745	0.7408	0.027*	
H9B	0.0903	1.0675	0.7194	0.027*	
C10	0.04473 (18)	0.90619 (14)	0.84323 (10)	0.0219 (2)	
C11	0.20475 (18)	0.90263 (15)	0.89814 (10)	0.0246 (2)	
H11A	0.2826	0.9654	0.8689	0.030*	
C12	0.2537 (2)	0.80858 (16)	0.99563 (11)	0.0303 (3)	
C13	0.1374 (2)	0.71844 (17)	1.03722 (12)	0.0348 (3)	
H13A	0.1688	0.6535	1.1033	0.042*	
C14	−0.0235 (2)	0.72173 (17)	0.98378 (12)	0.0347 (3)	
H14A	−0.1020	0.6597	1.0137	0.042*	
C15	−0.0708 (2)	0.81538 (16)	0.88651 (11)	0.0287 (3)	
H15A	−0.1813	0.8174	0.8498	0.034*	
C20	0.4298 (2)	0.8049 (2)	1.05269 (14)	0.0455 (4)	
H20A	0.5070	0.6990	1.0692	0.068*	
H20B	0.3894	0.8551	1.1224	0.068*	
H20C	0.5076	0.8584	1.0037	0.068*	

### Atomic displacement parameters (Å^2^)

**Table:**

	*U*^11^	*U*^22^	*U*^33^	*U*^12^	*U*^13^	*U*^23^
S1	0.01938 (14)	0.02259 (14)	0.02136 (14)	−0.01061 (11)	0.00229 (10)	−0.00472 (10)
Cl1	0.02011 (15)	0.03273 (17)	0.03346 (17)	−0.01163 (12)	0.00420 (11)	−0.00644 (12)
O1	0.0204 (4)	0.0311 (5)	0.0311 (5)	−0.0101 (4)	0.0026 (3)	−0.0085 (4)
O2	0.0231 (4)	0.0243 (4)	0.0412 (5)	−0.0111 (4)	0.0024 (4)	−0.0034 (4)
N1	0.0190 (4)	0.0195 (4)	0.0197 (4)	−0.0079 (4)	0.0003 (3)	−0.0030 (4)
N2	0.0192 (5)	0.0215 (5)	0.0203 (4)	−0.0089 (4)	0.0008 (4)	−0.0049 (4)
C1	0.0190 (5)	0.0172 (5)	0.0181 (5)	−0.0056 (4)	−0.0012 (4)	−0.0004 (4)
C2	0.0175 (5)	0.0202 (5)	0.0221 (5)	−0.0054 (4)	−0.0003 (4)	−0.0004 (4)
C3	0.0225 (6)	0.0219 (5)	0.0260 (6)	−0.0035 (4)	0.0021 (4)	−0.0055 (4)
C4	0.0288 (6)	0.0209 (5)	0.0293 (6)	−0.0066 (5)	0.0007 (5)	−0.0081 (5)
C5	0.0247 (6)	0.0220 (5)	0.0269 (6)	−0.0103 (5)	0.0003 (4)	−0.0052 (4)
C6	0.0197 (5)	0.0193 (5)	0.0199 (5)	−0.0069 (4)	0.0005 (4)	−0.0017 (4)
C7	0.0189 (5)	0.0181 (5)	0.0177 (5)	−0.0074 (4)	−0.0013 (4)	−0.0009 (4)
C8	0.0206 (5)	0.0181 (5)	0.0200 (5)	−0.0045 (4)	−0.0007 (4)	−0.0005 (4)
C9	0.0256 (6)	0.0188 (5)	0.0213 (5)	−0.0046 (4)	0.0018 (4)	−0.0039 (4)
C10	0.0235 (5)	0.0200 (5)	0.0197 (5)	−0.0034 (4)	0.0041 (4)	−0.0048 (4)
C11	0.0240 (6)	0.0243 (6)	0.0235 (5)	−0.0047 (5)	0.0032 (4)	−0.0052 (4)
C12	0.0282 (6)	0.0319 (7)	0.0245 (6)	−0.0002 (5)	0.0015 (5)	−0.0032 (5)
C13	0.0387 (8)	0.0317 (7)	0.0259 (6)	−0.0027 (6)	0.0045 (5)	0.0040 (5)
C14	0.0375 (8)	0.0315 (7)	0.0327 (7)	−0.0120 (6)	0.0100 (6)	0.0018 (5)
C15	0.0274 (6)	0.0299 (6)	0.0284 (6)	−0.0096 (5)	0.0044 (5)	−0.0032 (5)
C20	0.0377 (8)	0.0574 (11)	0.0365 (8)	−0.0071 (8)	−0.0108 (6)	0.0042 (7)

### Geometric parameters (Å, °)

**Table:**

S1—C6	1.7382 (12)	C5—H5A	0.9500
S1—C7	1.7436 (12)	C8—C9	1.5144 (16)
Cl1—C2	1.7310 (12)	C9—C10	1.5186 (17)
O1—C8	1.2250 (15)	C9—H9A	0.9900
O2—H2OB	0.876 (13)	C9—H9B	0.9900
O2—H2OA	0.883 (13)	C10—C11	1.3866 (18)
N1—C7	1.3068 (15)	C10—C15	1.3934 (18)
N1—C1	1.3884 (14)	C11—C12	1.3963 (18)
N2—C8	1.3633 (15)	C11—H11A	0.9500
N2—C7	1.3809 (14)	C12—C13	1.387 (2)
N2—H2N	0.868 (13)	C12—C20	1.504 (2)
C1—C2	1.4004 (15)	C13—C14	1.383 (2)
C1—C6	1.4028 (16)	C13—H13A	0.9500
C2—C3	1.3799 (17)	C14—C15	1.391 (2)
C3—C4	1.3964 (18)	C14—H14A	0.9500
C3—H3A	0.9500	C15—H15A	0.9500
C4—C5	1.3853 (17)	C20—H20A	0.9800
C4—H4A	0.9500	C20—H20B	0.9800
C5—C6	1.3932 (16)	C20—H20C	0.9800
			
C6—S1—C7	88.18 (5)	C8—C9—C10	108.76 (10)
H2OB—O2—H2OA	107.2 (14)	C8—C9—H9A	109.9
C7—N1—C1	109.19 (10)	C10—C9—H9A	109.9
C8—N2—C7	123.21 (10)	C8—C9—H9B	109.9
C8—N2—H2N	118.6 (10)	C10—C9—H9B	109.9
C7—N2—H2N	118.0 (11)	H9A—C9—H9B	108.3
N1—C1—C2	126.10 (11)	C11—C10—C15	119.52 (12)
N1—C1—C6	115.52 (10)	C11—C10—C9	119.83 (11)
C2—C1—C6	118.38 (11)	C15—C10—C9	120.63 (12)
C3—C2—C1	120.19 (11)	C10—C11—C12	121.27 (13)
C3—C2—Cl1	120.05 (9)	C10—C11—H11A	119.4
C1—C2—Cl1	119.75 (9)	C12—C11—H11A	119.4
C2—C3—C4	120.06 (11)	C13—C12—C11	118.40 (14)
C2—C3—H3A	120.0	C13—C12—C20	121.33 (14)
C4—C3—H3A	120.0	C11—C12—C20	120.26 (14)
C5—C4—C3	121.51 (11)	C14—C13—C12	120.94 (13)
C5—C4—H4A	119.2	C14—C13—H13A	119.5
C3—C4—H4A	119.2	C12—C13—H13A	119.5
C4—C5—C6	117.62 (11)	C13—C14—C15	120.30 (14)
C4—C5—H5A	121.2	C13—C14—H14A	119.8
C6—C5—H5A	121.2	C15—C14—H14A	119.8
C5—C6—C1	122.21 (11)	C14—C15—C10	119.57 (13)
C5—C6—S1	128.07 (9)	C14—C15—H15A	120.2
C1—C6—S1	109.71 (8)	C10—C15—H15A	120.2
N1—C7—N2	120.77 (10)	C12—C20—H20A	109.5
N1—C7—S1	117.35 (9)	C12—C20—H20B	109.5
N2—C7—S1	121.88 (8)	H20A—C20—H20B	109.5
O1—C8—N2	121.69 (11)	C12—C20—H20C	109.5
O1—C8—C9	122.33 (11)	H20A—C20—H20C	109.5
N2—C8—C9	115.92 (10)	H20B—C20—H20C	109.5
			
C7—N1—C1—C2	−179.68 (11)	C8—N2—C7—N1	175.97 (11)
C7—N1—C1—C6	0.58 (14)	C8—N2—C7—S1	−5.13 (16)
N1—C1—C2—C3	178.77 (11)	C6—S1—C7—N1	2.23 (10)
C6—C1—C2—C3	−1.50 (17)	C6—S1—C7—N2	−176.71 (10)
N1—C1—C2—Cl1	−2.55 (17)	C7—N2—C8—O1	−4.83 (18)
C6—C1—C2—Cl1	177.18 (9)	C7—N2—C8—C9	172.42 (10)
C1—C2—C3—C4	0.67 (19)	O1—C8—C9—C10	81.73 (14)
Cl1—C2—C3—C4	−178.01 (10)	N2—C8—C9—C10	−95.49 (12)
C2—C3—C4—C5	0.5 (2)	C8—C9—C10—C11	114.28 (12)
C3—C4—C5—C6	−0.8 (2)	C8—C9—C10—C15	−63.84 (14)
C4—C5—C6—C1	−0.11 (19)	C15—C10—C11—C12	0.61 (18)
C4—C5—C6—S1	179.87 (10)	C9—C10—C11—C12	−177.53 (11)
N1—C1—C6—C5	−179.00 (11)	C10—C11—C12—C13	−0.31 (19)
C2—C1—C6—C5	1.24 (18)	C10—C11—C12—C20	179.05 (13)
N1—C1—C6—S1	1.01 (13)	C11—C12—C13—C14	−0.2 (2)
C2—C1—C6—S1	−178.75 (9)	C20—C12—C13—C14	−179.55 (14)
C7—S1—C6—C5	178.34 (12)	C12—C13—C14—C15	0.4 (2)
C7—S1—C6—C1	−1.67 (9)	C13—C14—C15—C10	−0.1 (2)
C1—N1—C7—N2	176.95 (10)	C11—C10—C15—C14	−0.39 (19)
C1—N1—C7—S1	−2.00 (13)	C9—C10—C15—C14	177.73 (12)

### Hydrogen-bond geometry (Å, °)

**Table:**

*D*—H···*A*	*D*—H	H···*A*	*D*···*A*	*D*—H···*A*
O2—H2OB···N1^i^	0.88 (1)	2.10 (2)	2.924 (1)	158 (2)
O2—H2OA···O1^ii^	0.88 (1)	2.05 (1)	2.904 (1)	164 (2)
N2—H2N···O2	0.87 (1)	1.92 (1)	2.785 (1)	177 (2)
C5—H5A···O1^iii^	0.95	2.56	3.351 (2)	141.
C3—H3A···Cg3^iv^	0.95	2.66	3.502 (1)	148

Symmetry codes: (i) −*x*+1, −*y*+2, −*z*+1; (ii) *x*+1, *y*, *z*; (iii) −*x*, −*y*+1, −*z*+1; (iv) −*x*+1, −*y*+1, −*z*+1.

### Table 2 Cg···Cg π-stacking interactions, Cg1, Cg2 and Cg3 are the centroids of rings S1/C6/C1/N1/C7 C1–C6 and C10–C15, respectively. [Symmetry codes: (i) 1-x, -1-y, 1-z; (ii) -x, 2-y, 2-z]

**Table:**

*Cg*I···*Cg*J	CgI···CgJ (Å)	CgI···Perp (Å)	CgJ···Perp (Å)
Cg1···Cg1^i^	3.8743 (7)	3.5381 (4)	3.581 (4)
Cg1···Cg2^i^	3.7229 (7)	3.5474 (4)	3.5229 (5)
Cg3···Cg3^ii^	3.7076 (8)	3.4878 (6)	3.4878 (6)
